# Multisite, multimodal neuroimaging of chronic urological pelvic pain: Methodology of the MAPP Research Network

**DOI:** 10.1016/j.nicl.2015.12.009

**Published:** 2016-01-06

**Authors:** Jeffry R. Alger, Benjamin M. Ellingson, Cody Ashe-McNalley, Davis C. Woodworth, Jennifer S. Labus, Melissa Farmer, Lejian Huang, A. Vania Apkarian, Kevin A. Johnson, Sean C. Mackey, Timothy J. Ness, Georg Deutsch, Richard E. Harris, Daniel J. Clauw, Gary H. Glover, Todd B. Parrish, Jan den Hollander, John W. Kusek, Chris Mullins, Emeran A. Mayer, the MAPP Research Network Investigators

**Affiliations:** a Department of Neurology, David Geffen School of Medicine at UCLA, USA; b Department of Radiological Sciences, David Geffen School of Medicine at UCLA, USA; c Department of Biomedical Physics, David Geffen School of Medicine at UCLA, USA; d UCLA Brain Research Institute, David Geffen School of Medicine at UCLA, USA; e UCLA Ahmanson-Lovelace Brain Mapping Center, David Geffen School of Medicine at UCLA, USA; f NeuroSpectroScopics, LLC, Sherman Oaks, CA, USA; g Department of Internal Medicine, David Geffen School of Medicine at UCLA, USA; h Oppenheimer Center for Neurobiology of Stress at UCLA, David Geffen School of Medicine at UCLA, USA; i Department of Bioengineering, Henry Samueli School of Engineering and Applied Science at UCLA, USA; j Department of Psychiatry and Biobehavioral Sciences, David Geffen School of Medicine at UCLA, USA; k Department of Physiology, Feinberg School of Medicine, Northwestern University, 300 E. Superior St., Chicago, IL 60611, USA; l Departments of Anesthesia and Surgery, Feinberg School of Medicine, Northwestern University, 300 E. Superior St., Chicago, IL 60611, USA; m Department of Neurology, Stanford University, Palo Alto, CA, USA; n Department of Anesthesiology, University of Alabama, Birmingham, AL, USA; o Department of Radiology, University of Alabama, Birmingham, AL, USA; p Department of Anesthesiology, University of Michigan, Ann Arbor, MI, USA; q Department of Radiology, Stanford University, Palo Alto, CA, USA; r Department of Radiology, Northwestern University, Chicago, IL, USA; s Department of Radiology, University of Alabama at Birmingham, Birmingham, AL, USA; t Division of Kidney, Urologic, and Hematologic Diseases, National Institute of Diabetes and Digestive and Kidney Diseases, National Institutes of Health, Bethesda, MD, USA

**Keywords:** Urologic chronic pelvic pain syndromes, Brain, Magnetic resonance imaging, Functional magnetic resonance imaging, Diffusion tensor imaging, DTI, TransMAPP

## Abstract

The Multidisciplinary Approach to the Study of Chronic Pelvic Pain (MAPP) Research Network is an ongoing multi-center collaborative research group established to conduct integrated studies in participants with urologic chronic pelvic pain syndrome (UCPPS). The goal of these investigations is to provide new insights into the etiology, natural history, clinical, demographic and behavioral characteristics, search for new and evaluate candidate biomarkers, systematically test for contributions of infectious agents to symptoms, and conduct animal studies to understand underlying mechanisms for UCPPS. Study participants were enrolled in a one-year observational study and evaluated through a multisite, collaborative neuroimaging study to evaluate the association between UCPPS and brain structure and function. 3D T1-weighted structural images, resting-state fMRI, and high angular resolution diffusion MRI were acquired in five participating MAPP Network sites using 8 separate MRI hardware and software configurations. We describe the neuroimaging methods and procedures used to scan participants, the challenges encountered in obtaining data from multiple sites with different equipment/software, and our efforts to minimize site-to-site variation.

## Highlights

• The MAPP Research Network has implemented a multi-site, multi-modal, MRI protocol.
• Despite initial challenges, the protocol was standardized across sites.
• Even with near-identical protocols, site differences were present in the data.
• Appropriate site-correction methods have led to multiple neuroimaging publications.
• Improved site harmonization is being implemented in the second phase of the study.

## 1 Introduction

The Multidisciplinary Approach to the Study of Chronic Pelvic Pain (MAPP) Research Network was established by the National Institute of Diabetes and Digestive and Kidney Diseases in 2008 to conduct collaborative and integrated studies in men and women with urologic chronic pelvic pain syndrome (UCPPS), a term which includes interstitial cystitis/bladder pain syndrome (IC/BPS) and chronic prostatitis/chronic pelvic pain syndrome (CP/CPPS) (Clemens et al., 2014, Landis et al., 2014). The Network's goals are to identify clinically relevant phenotypes for these syndromes, their underlying pathophysiology, describe the treated natural history; and identify biologic, genetic, and behavioral risk factors for UCPPS. A subgroup of study participants enrolled in a one-year prospective observational study, the trans-MAPP Epidemiology and Phenotyping Study, had their brain structure and function evaluated with advanced neuroimaging techniques. Healthy controls and persons with irritable bowel syndrome, chronic fatigue syndrome and fibromyalgia (“positive controls”) were also studied.

The MAPP Neuroimaging Study utilized three magnetic resonance imaging (MRI) technologies, each aimed at providing unique information regarding brain structure or function. Structural brain imaging was performed using 3D T1-weighted imaging to provide high contrast between various tissue types (e.g. gray matter (GM) and white matter (WM)) to evaluate associations between brain morphometry and a wide array of characteristics of these syndromes. The associations between brain network communications and UCPPS was investigated with resting-state functional MRI using time resolved multislice T2*-weighted echo planar imaging (EPI). Diffusion tensor imaging (DTI) was also performed with multi-slice diffusion-weighted spin echo EPI to assess the relationship between WM microstructural alterations associated with UCPPS. We hypothesize that when combined with simultaneously acquired clinical, behavioral, and biological data, obtained through the trans-MAPP EP Study, these three advanced structural and functional neuroimaging techniques performed on a large number of participants from five different network sites will not only inform about the pathophysiology of UCPPS, but may also help to identify clinical useful imaging biomarkers and distinct phenotypic profiles associated with UCPPS. It is expected that these findings will inform both clinical management and future clinical trials.

We describe the specific neuroimaging methodologies used in our multi-site study and provide summaries of quality assurance, quality control, and image processing procedures that were used to assess the MAPP Network neuroimaging data.

## 2 Subjects and methods

The MAPP Network Neuroimaging Study involved a total of five different institutions with various hardware and software configurations. Pulse sequence parameters were standardized across sites to the extent permitted by each platform. Neuroimaging procedures were documented and distributed to participating centers to provide them with targeted parameters and methods for image acquisition (manual of procedures is available at http://painrepository.org/documents/transmapp/manuals/TransMAPP_MRIProceduresManual_20110224.pdf). Neuroimaging was performed at the medical center campuses of Northwestern University (NW), Stanford University (Stanford), University of Alabama at Birmingham (UAB), University of California — Los Angeles (UCLA), and the University of Michigan (Michigan). All centers used 3 T high-performance MR scanners for image acquisition from one of three manufacturers: Siemens Healthcare (Siemens), GE Medical Systems (GEMS), or Philips Healthcare (Philips). Specific MRI system details and software configurations are shown in Table 2, Table 3, Table 4. Note that several centers underwent software upgrades over the course of the study and several sites with similar scanners had slightly different software configurations.

### 2.1 Overview of trans-MAPP EP Study participants

The organization of the MAPP Research Network and the design of the trans-MAPP Epidemiology and Phenotyping Study have been described in detail previously (Landis et al., 2014, Clemens et al., 2014). A total of 329 of 1039 participants enrolled in the study received a scan at baseline. They included 132 persons with UCPPS, 128 healthy controls, 69 sex and age-matched “positive” controls including participants with irritable bowel syndrome (IBS) and fibromyalgia (Table 1). All MRI scans from the 6 discovery sites included 3D T1-weighted structural and resting state functional MRI (rs-fMRI) sequences. Additionally five sites acquired diffusion tensor imaging (DTI) MRI.

### 2.2 3D T1-weighted structural MRI

A magnetization prepared rapid gradient echo (MP-RAGE) pulse sequence was used for high-resolution, 3D T1-weighted structural MRI in Siemens and Philips scanners, while an inversion-recovery fast spoiled gradient echo (IR-FSPGR) sequence was used for 3D T1-weighted structural MRI on GEMS scanners at one site. This pulse sequence has been standardized across manufacturers and software platforms as part of the Alzheimer's Disease Neuroimaging Initiative (ADNI) (Jack et al., 2004), which used the MP-RAGE and IR-FSPGR sequences as the primary structural imaging method. MP-RAGE/IR-FSPGR sequences provide excellent tissue contrast at nearly isotropic spatial resolution around 1 mm^3^. Table 2 illustrates the particular pulse sequence timing and acquisition parameters used at each site. It is important to note that GEMS platforms do not have the particular MP-RAGE pulse sequence available, therefore, the comparable IR-FSPGR sequence was used, which has a different definition for certain pulse sequence parameters including repetition time (TR).

### 2.3 Resting-state fMRI

Resting-state fMRI (rs-fMRI) acquisition parameters followed recommendations from the functional bioinformatics research network (fBIRN) (Glover et al., 2012), and are described in Table 3. Briefly, our rs-fMRI acquisition protocol used a target run length of 10 min to enhance for filtering of low frequency fluctuations from raw temporal data.

### 2.4 Diffusion tensor imaging

Diffusion tensor images (DTI) were acquired at four of the participating centers. Our DTI protocol included diffusion-weighted image acquisition in at least 32 diffusion sensitizing directions (ideally > 60 directions) and a maximum *b*-value of 1000 s/mm^2^ to enable probabilistic tractography and other sophisticated post-processing techniques that require relatively high angular resolution diffusion imaging. Hardware and software differences across MRI systems made homogeneous acquisition of DTI data challenging, particularly at centers without the ability to modify the diffusion direction information directly. These differences were due to inherent differences in diffusion timing relating to gradient performance, maximum *b*-values limited by gradient strength, and the ability to prescribe particular diffusion-sensitizing directions. Despite these efforts, variability in acquisition parameters across participating sites was inherent in the DTI data. Table 4 lists the DTI pulse sequence timing and acquisition parameters used at each site.

### 2.5 Image upload/download

DICOM formatted image data were uploaded from each participating site to the Image Data Archive operated by the University of Southern California (USC) Laboratory of NeuroImaging (LONI) (http://ida.loni.usc.edu). In addition to providing archive services, LONI also provides format conversion services to permit Network investigators to download images in NIFTI or DICOM format.

### 2.6 Quality assurance and quality control

Each participating MAPP Network imaging site collected a series of phantom images to ensure adequate image quality. Structural MR images of the ADNI phantom were collected at each center using pre-prescribed image acquisition and pulse sequence settings (see MAPP neuroimaging procedures manual http://painrepository.org/documents/transmapp/manuals/TransMAPP_MRIProceduresManual_20110224.pdf). In certain circumstances, sites were allowed to submit equivalent phantom data (e.g. the American College of Radiology (ACR) standardized phantom). The ADNI phantom object images were visually inspected to identify gross geometric distortions in structural MR data. The sites were also required to obtain and perform a series of rs-fMRI studies on the fBIRN phantom to ensure temporal stability of the sequence (Friedman and Glover, 2006a, Friedman and Glover, 2006b). Sites collected rs-fMRI data on the fBIRN phantom on the same day as participants for the first 10 scans or until temporal stability was confirmed. Quality assurance of phantom data compared static spatial noise variance (SNR), signal drift and fluctuation, and Fourier analysis of residuals. Metrics from each site complied with defined ranges and recommendations (Glover et al., 2012). Quality control procedures for DTI human data evaluated ranges of apparent diffusion coefficient (ADC) and fractional anisotropy (FA) for various reference structures (e.g. cerebrospinal fluid and normal white matter), examining color-coded FA maps for adequate tensor directionality, assessment of bulk motion artifacts, and assessment of susceptibility-related geometric distortions. In particular ADC values in WM and ventricular cerebrospinal fluid were required to be within a range of 0.5–0.8 μm^2^/ms and 2.5–3.5 μm^2^/ms, respectively, while FA values in the corpus callosum and cerebrospinal fluid were required to be within a range of 0.6–1.0 and 0.0–0.2, respectively.

Each MRI data acquisition uploaded to the LONI repository underwent a quality control evaluation that was not designed to reject studies, but rather flag acquisition errors and other technical problems so that investigators who might use these data in the future would have annotated images. Structural MRI data were assessed visually for subject motion artifacts, poor image contrast, and errors in image prescription. Rs-fMRI data were examined for motion using the fBIRN quality control workflow (BXH/XCEDE tools), which produces a full report including SNR and signal fluctuation to noise ratio (SFNR) along with metrics of global head motion, signal drift, and spurious fluctuations. DTI data were flagged for variations in quantitative metrics (e.g. ADC and FA), motion artifacts, gradient failures, and geometric distortions.

### 2.7 Selection of subgroups for center comparisons

Data from the MAPP Network were used to describe MR measurement variations across centers. All healthy control females aged 20–35 provided a data set consisting of 47 different subjects (6 from NW, 11 from UCLA, 14 from Michigan, 6 from UAB, 10 from Stanford) for structural MRI and rs-fMRI datasets. A subset of 27 healthy control females (5 from NW, 5 from UCLA, 5 from UAB, 12 from Stanford) was used for DTI analysis. Additionally, an rs-fMRI analysis was performed using all healthy control, UCPPS, and positive control participants' neuroimaging data acquired by January 2013 with a total of 263 participants (52 from NW, 85 from UCLA, 60 from Michigan, 41 from Stanford, 25 from UAB), and a DTI analysis utilized all healthy controls that underwent DTI scanning with a total 94 subjects (28 from NW, 31 from UCLA, 26 from Stanford, and 12 from UAB).

### 2.8 Image processing and statistical analyses

#### 2.8.1 Structural MRI

Structural MRI data obtained from the normal control female 20–35 years of age cohort underwent N3 intensity normalization, brain extraction using the Brain Extraction Tool (BET), and a 4 component (background, cerebrospinal fluid, GM, and WM) fuzzy *c*-means tissue segmentation using MIPAV software with default parameter settings. Failures in segmentation were identified by visual inspection. Custom software to measure whole brain GM and WM volumes from each study were implemented in Interactive Data Language (IDL). A Kuskal–Wallis test was used to identify statistically significant differences in whole brain WM and GM volumes across centers.

In addition to this subset, MAPP Network neuroscan data from 293 participants across all sites were evaluated independently using a custom-designed quality control pipeline that checks headers, extracts skull, and registers it to standard space. Across-center differences in brain tissue volume (normalized for subject head size) were further identified with Structural Image Evaluation using Normalization of Atrophy Cross-Sectional (SIENAX) derived estimates of peripheral GM (*pgray*) (Smith et al., 2002). We sought to define a single algorithm that could be used to statistically adjust for site differences in brain structural data (including DTI, see below) that accounted for within- and between-site variability. The linear correction that resolved between-center differences in mean and variability was identified for each subject as follows:

$$\mathrm{cf}=\left(x_{n}–{\overset{̅}{x}}_{c}\right)+{\overset{̅}{x}}_{\mathrm{tot}}$$

where cf is an individual's correction factor, *x*_*n*_ is subject's uncorrected *pgray* value, ${\overset{̅}{x}}_{c}$ is mean *pgray* value of subject's site, and ${\overset{̅}{x}}_{\mathrm{tot}}$ is grand mean *pgray* across all sites. This correction adjusts for individual site effect and adds this corrected value to the grand mean of all sites.

#### 2.8.2 rs-fMRI

Quality control evaluations were performed on the rs-fMRI data using fBIRN software tools and the normal control female 20–35 years of age data as described by Friedman et al. (Friedman and Glover, 2006a, Friedman and Glover, 2006b). Additionally, data from 293 healthy volunteers, UCPPS, and positive control participants were preprocessed using FSL's FMRI Expert Analysis Tool (FEAT) version 5.98, including skull extraction, slice-timing correction, head motion correction, spatial smoothing (with a Gaussian kernel of full-width-half-maximum 5 mm), and a high-pass (150 s) temporal filter. Independent components analysis (ICA) implemented with the MELODIC tool in FSL, and time courses of cerebral spinal fluid and WM single-voxel regions of interest (ROIs) were regressed out of the BOLD signal, as well as the global mean BOLD signal. Voxel-wise functional correlation matrices were generated using pair-wise Pearson correlations, corrected for distance between regions, and thresholded over a range of connection strengths. These connection strengths, or link densities, represent the percentage of connections in a correlation matrix that exceed given correlation thresholds that are consistent with small-world topology (detailed methods in Baria et al., 2013). Using a connection density of 0.1, a mean map of voxel-wise degree across all subjects to test for center, age, and gender effects was generated (corrected for positive false discovery rate, pFDR). Given the absence of standards for across-site compatibility of rs-fMRI data, we compared default mode network properties, which have been extensively studied in healthy populations and in persons with pain, to estimate similarity of information flow across participant groups and sites (corrected for pFDR).

#### 2.8.3 DTI

DTI data from the entire healthy control cohort (94 subjects) were first eddy current and distortion corrected using FSL's Diffusion Toolbox (http:/fsl.fmrib.ox.ac.uk/fsl/fslwiki/FDT) using the initial *b* = 0 s/mm^2^ volume as the reference. ADC and FA images were calculated using the MRtrix software package (http://www.brain.org.au/software/mrtrix). Only diffusion data with ADC and FA maps that passed quality assurance were included in this analysis. FA images for each participant were registered to the Johns Hopkins University DTI atlas (ICBM-DTI-81 1 mm FA atlas) using a 12-degree of freedom linear affine transformation in FSL. After linear registration, elastic (nonlinear) registration was performed between individual FA maps and the ICBM-DTI-8 1 mm FA atlas using the FNIRT command in FSL. The transformation matrices (linear, then nonlinear) were then used to align ADC maps to the same atlas space. WM voxels were defined using an FA threshold of 0.3 and above. Deep gray matter structures were also included in the mask for analysis. Statistical parameter mapping was performed using a general linear model in AFNI to identify spatially-specific differences in WM FA and ADC across sites. Age and gender information were used as covariates in the GLM. Statistical significance was defined as *P* < 0.05 using a false discovery rate q < 0.05 and a minimum cluster size of 250 μL. In addition, mean values from spherical regions of interest (ROIs) (4 mm radius) placed on the corpus callosum (CC) and a WM region in the superior corona radiata were measured. A one-way ANOVA test was performed with Tukey's test for multiple comparisons to examine differences in ROI-based FA and ADC measurements across centers. For subset analyses in the normal control female 20–35 years of age, mean ADC, FA, axial diffusivity (AD, defined as the diffusivity along the principal eigenvector orientation) and radial diffusivity (RD, defined as the diffusivity perpendicular to the principal eigenvector orientation) were calculated in IDL and evaluated across various ROIs.

Given the possibility that selected ROIs contained a combination of WM and GM, we then calculated the mean skeletal FA for each subject using a common WM skeleton template derived from pooled participant groups. Mean FA values for each center were contrasted using permutation methods (*n* = 5000 permutations, *P* < 0.05). A one-way ANOVA was used to compare mean FA values across centers. The linear correction method outlined for structural 3D data was also used for DTI data.

## 3 Results

### 3.1 Phantom assessment

**Fig. 1:**
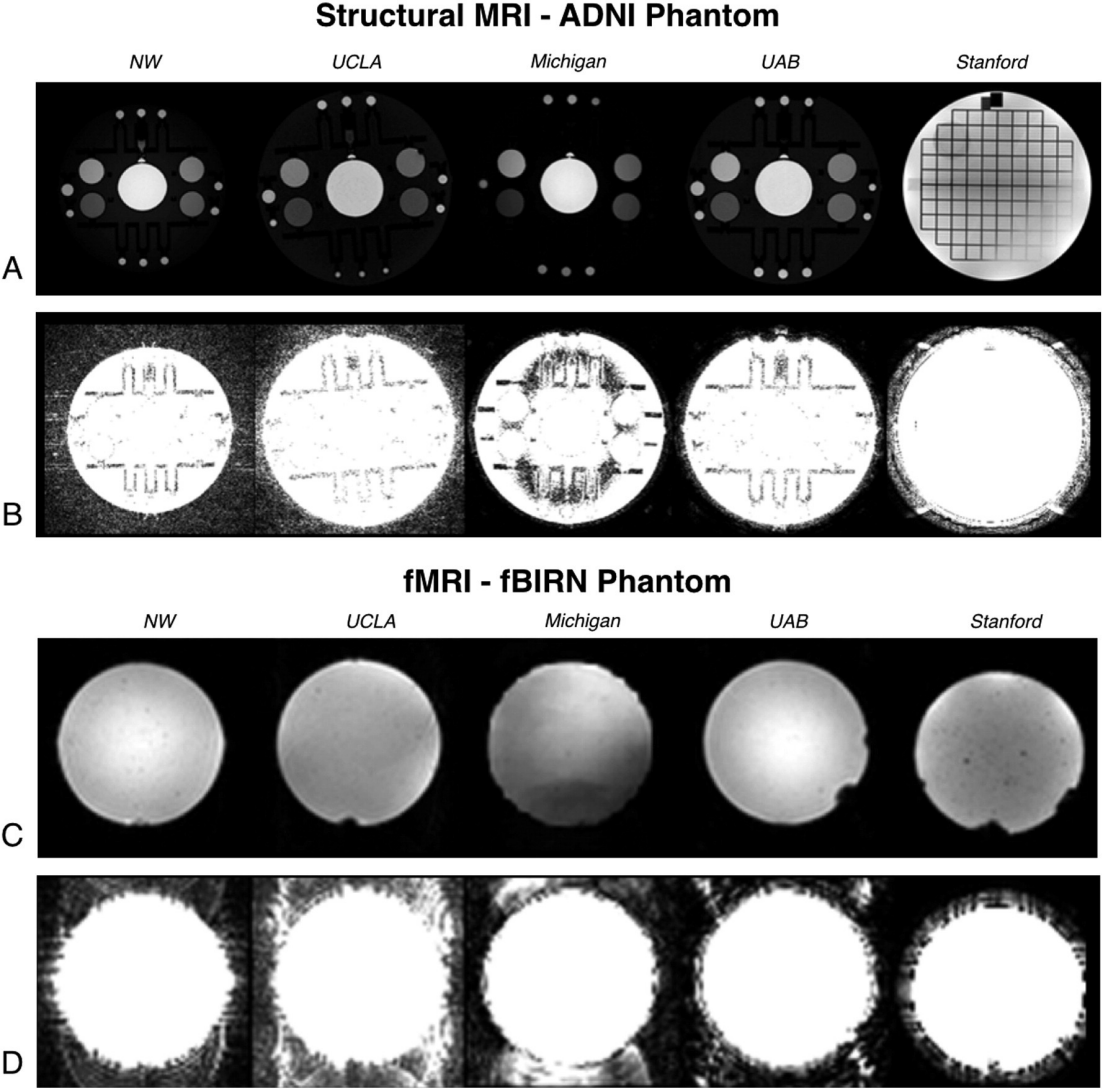
Structural and fMRI phantom results. A) Center slice from axial 3D T1-weighted (MPRAGE or IR-FSPGR) images from ADNI (or ACR) phantom (intensity autoscaled). B) Equivalent slices from (A) upscaled 100-fold to permit visualization of background noise and artifacts. C) Representative resting-state fMRI images of the fBIRN phantom (intensity autoscaled). D) Equivalent slices from (C) upscaled 100-fold to highlight background noise and artifacts.

Fig. 1 provides example images of the phantom objects used at the participating MAPP Network sites. As illustrated by this figure, the MRI MR systems at each site used slightly different procedures for digitally representing MR signal intensity and noise characteristics. All systems used 16-bit digital image representation, however, absolute values and digital resolution appeared to differ across centers. The Siemens systems (NW and UCLA) produced DICOM images of the ADNI phantom objects that represented signal intensity as unsigned 16 bit integers using the range 0–2000 (11 bits). ADNI phantom object images collected using Philips systems represented images as 16 bit integers using a range of 0–850 (10 bits). GEMS systems recorded signal intensity as signed integers using a range of 0–24,000 (15 bits). In addition to this difference in digital representation, Philips and GEMS MRI systems used a symmetric Fermi *k*-space filter during image reconstruction that removed much of the background noise (Fig. 1B). The differences in digital scaling and reconstruction filtering are likely responsible for differences in contrast and background noise apparent in Fig. 1A–B). Because these filtering and post-processing steps for the raw-data are proprietary and vendor-specific, we opted to use the default settings at each scanner. While the variation in filtering techniques may decrease compatibility across scans, the presence of filtering in structural MRI is ubiquitous and individual configuration of filtering for maximum compatibility would have proven cumbersome. Thus, visual assessment of scan quality and reliable reconstruction and filtering on a site-by-site basis was preferred.

Examination of the rs-fMRI data collected using the fBIRN phantom showed many of the same features as the structural MRI data, namely differences due to reconstruction filtering and digital resolution. Image uniformity was relatively consistent across centers, with one site illustrating slight variations in image intensity across the phantom (Fig. 1C). Similar to structural MR data, background noise was less apparent in data acquired using Philips MR systems compared with the Siemens systems. Interestingly, background noise was completely absent on GEMS systems, likely due to subsequent image filtering during post-processing (Fig. 1D). Background noise in the UCLA data appeared slightly higher than data acquired from NW. Furthermore, the N/2 aliasing artifacts present in the UCLA and NU data appear to differ slightly, with NW images illustrating horizontal streaking artifacts not present in UCLA data. These observations illustrate the slight differences in image quality across sites despite similar acquisition parameters and identical phantom objects.

### 3.2 Structural MRI

**Fig. 2:**
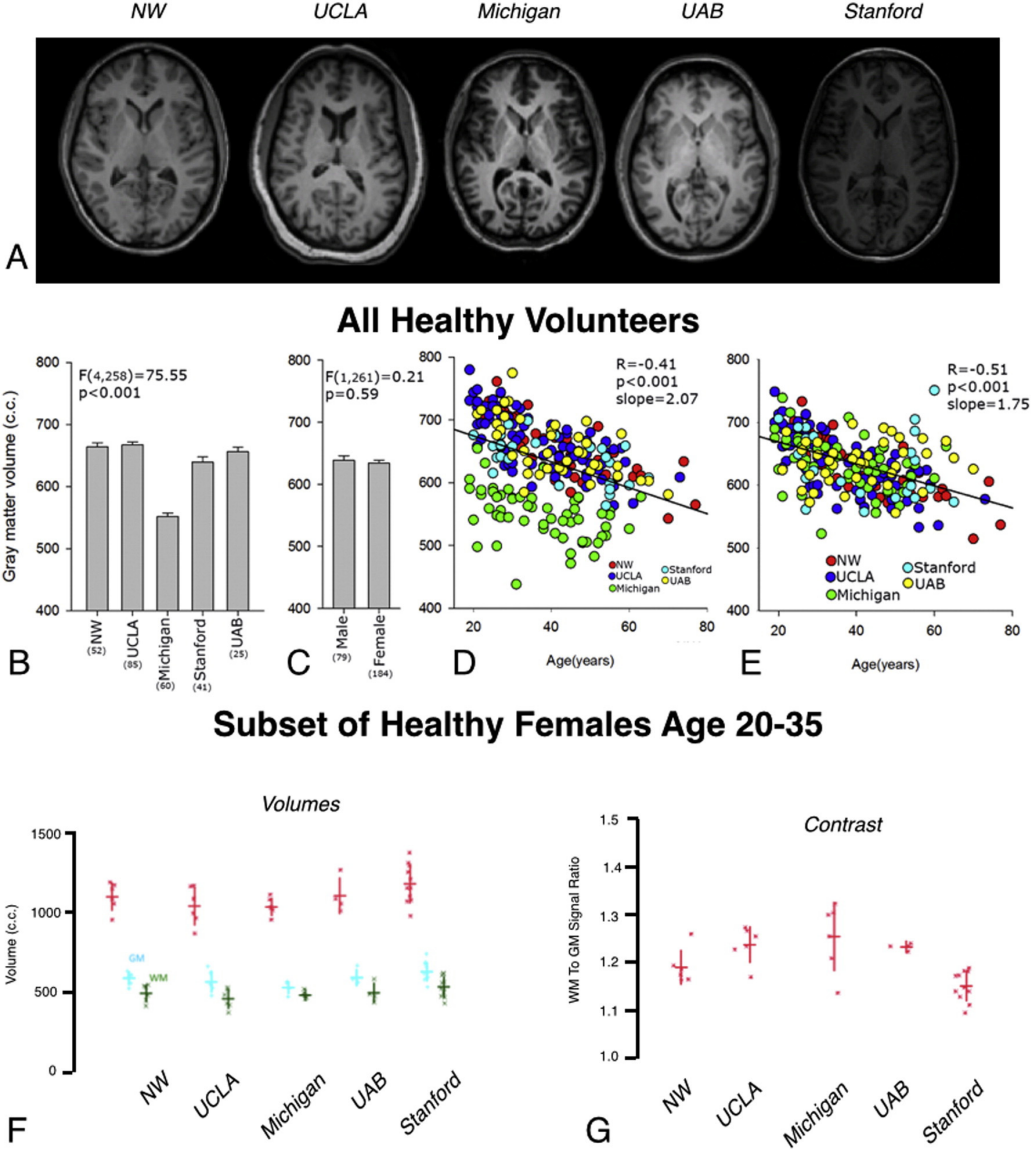
3D structural T1-weighted MR results. A) Representative 3D structural T1-weighted MR images of human subjects from each site at the level of the basal ganglia (intensity autoscaled). B) Peripheral GM volume (in cc) in healthy control subjects across the various sites, demonstrating a significantly lower GM volume at the University of Michigan compared with the other sites. C) Comparison of GM volume (in cc) between male and female healthy controls showing no significant difference. D) GM volume (in cc) appears to decrease linearly with age (green = Michigan; red = NW; dark blue = UCLA; light blue = Stanford; yellow = UAB). E) GM volume after correcting for site shows improved correlation as demonstrated by higher *R*^2^ (green = Michigan; red = NW; dark blue = UCLA; light blue = Stanford; yellow = UAB). F) Whole brain GM and WM volumes measured for each subject in the group of healthy control females age 20–35 stratified by site. Kruskal–Wallis *H*-test indicated a significant between site difference (*H* = 11.078, *P* = 0.0257) for GM volume but not for WM volume or the sum of WM and GM volume. (Note the difference is reduced substantially after exclusion of data from Michigan). Red = Total brain volume (GM + WM). Blue = GM volume. Green = WM volume. G) Ratio of WM to GM signal intensity (contrast) for each subject in the group of healthy control females separated by site. Kruskal–Wallis tests suggest a significant difference between sites with respect to GM to WM contrast (*H* = 16.071, *P* = 0.0029). For (F) and (G), horizontal lines reflect the group mean and vertical lines indicate one standard deviation above and below the mean. (For interpretation of the references to color in this figure legend, the reader is referred to the web version of this article.)

Consistent with phantom assessments, slight variations in image intensity were present in structural MRI scans across the various sites (Fig. 2A). Differences in peripheral GM volume were noted across sites, gender, and age. Results show that peripheral GM volume depends significantly on site (Fig. 2B; ANOVA, *P* < 0.001), with the University of Michigan showing a systematically lower volume compared with other sites. No differences in peripheral GM volume were found when comparing 293 male and female participants in this study (Fig. 2C; ANOVA, *P* = 0.59). An expected negative correlation was observed between peripheral GM volume and the age of the healthy controls (Fig. 2D; *R* = − 0.41, *P* < 0.001). This relationship was strengthened when correcting for both site and gender (Fig. 2E; *R* = − 0.51, *P* < 0.001). On average, peripheral GM volume decreased by 2 cm^3^ for each one year increase in age. Similar trends in brain volumes were observed in the analysis of a subset of healthy female controls between ages 20–35 years (Fig. 2F). In particular, no differences in WM volume or the sum of WM and GM volume were observed in this subset of healthy controls (Fig. 2F; Kruskal–Wallis, *P* > 0.05); however, there was a significant difference in GM volumes across sites (Kruskal–Wallis, *P* = 0.026). Lastly, quantitative estimates of tissue contrast between GM and WM were examined across centers in a subset of healthy controls (Fig. 2G). Results suggest a significant difference in the ratio of white to gray matter signal intensity across various centers (Fig. 2G; Kruskal–Wallis, *P* = 0.003).

### 3.3 Resting-state fMRI

**Fig. 3:**
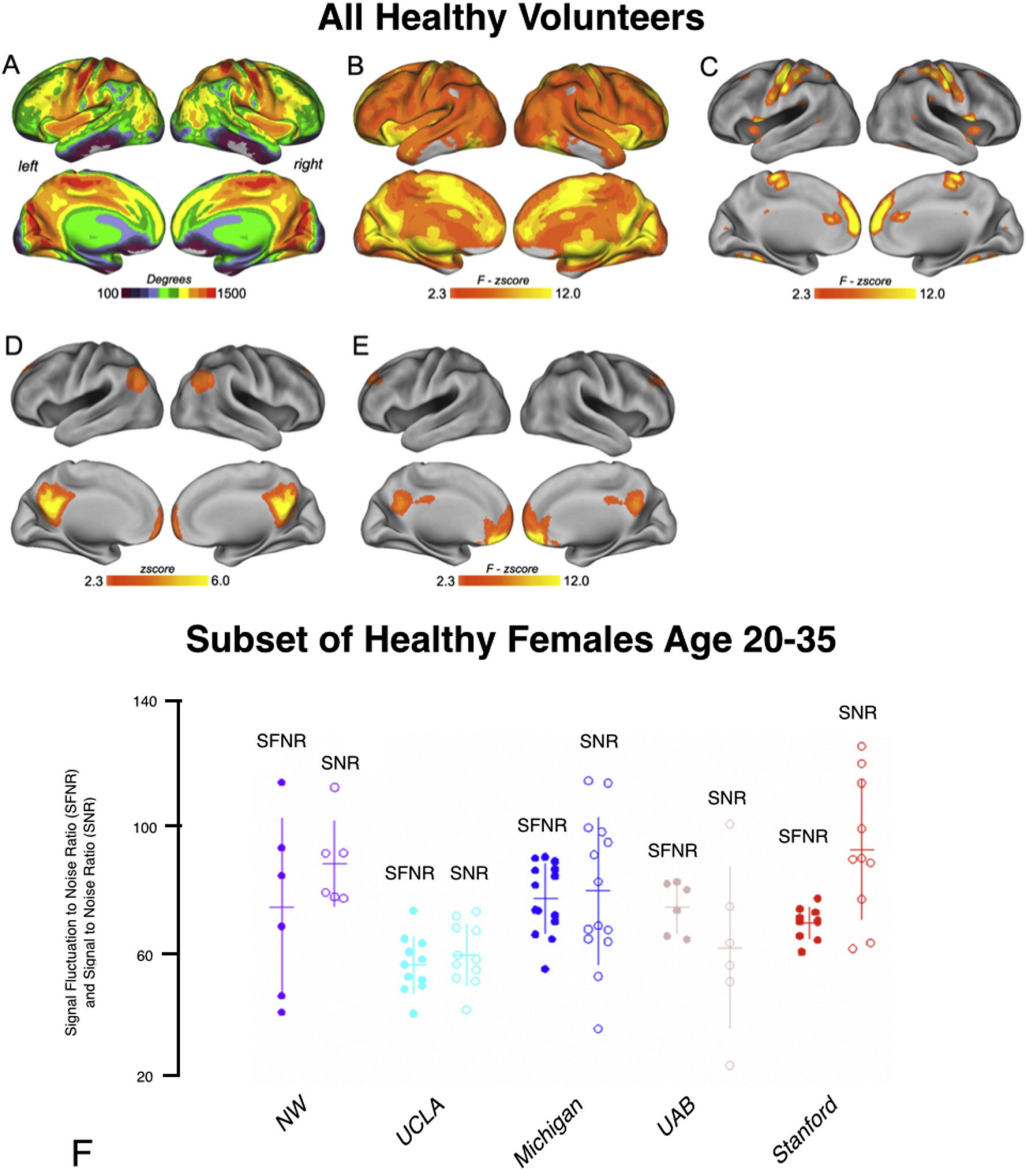
Resting-state fMRI results. A) Composite images depicting maps of mean voxel-wise degree of resting-state fMRI scans for link density of 0.1. B) Examination of the voxel-wise degree for rs-fMRI scans showing a strong and significant site effect even after pFDR correction for multiple comparisons. Degree maps illustrate a significant dependency on age, but no differences in degree distribution for gender. C) Regions with significant differences in mean degree maps as computed using a voxel-wise three way ANOVA with site, age, and gender as covariates. D) To determine the biological relevance of rs-fMRI connectivity across sites, the most reliably identified network in human neuroimaging—the default mode network, DMN—was identified per subject with voxel-wise independent components analysis and averaged across all subjects. E) Significant site differences in DMN connectivity were localized in medial prefrontal regions, with no main effects observed for age or gender. F) Signal-to-Fluctuation Noise Ratio (SFNR, filled circles) and Signal-to-Noise Ratio (SNR, open circles) from fBIRN analysis of the group of normal female control subjects stratified by site. Kruskal–Wallis tests suggest significant site differences for SFNR (*H* = 16.66, *P* = 0.002) and for SNR (*H* = 16.27, *P* = 0.027).

Composite images depicting the mean voxel-wise degree for 293 resting-state fMRI scans across all groups of participants scanned for link density of 0.1 are shown in Fig. 3A. Examination of the voxel-wise degree for rs-fMRI scans show a strong and significant site effect after correction for multiple comparisons using false positive discovery for the degree maps (Fig. 3B). Additionally, degree maps illustrated a significant dependency on age, but no differences in degree distribution for sex. Fig. 3C shows regions with significant differences in mean degree maps as computed using a voxel-wise three way ANOVA with site, age, and gender as covariates. A significant difference in default mode network (DMN) connectivity was observed across sites (Fig. 3D–E), but no significant differences were observed with respect to age or gender. Consistent with these observations, examination of SFNR and SNR in a subset of healthy female controls with age 20–35 showed significant differences across site (Fig. 3F; Kruskal–Wallis, *P* < 0.05 for both SFNR and SNR).

### 3.4 Diffusion tensor imaging

**Fig. 4:**
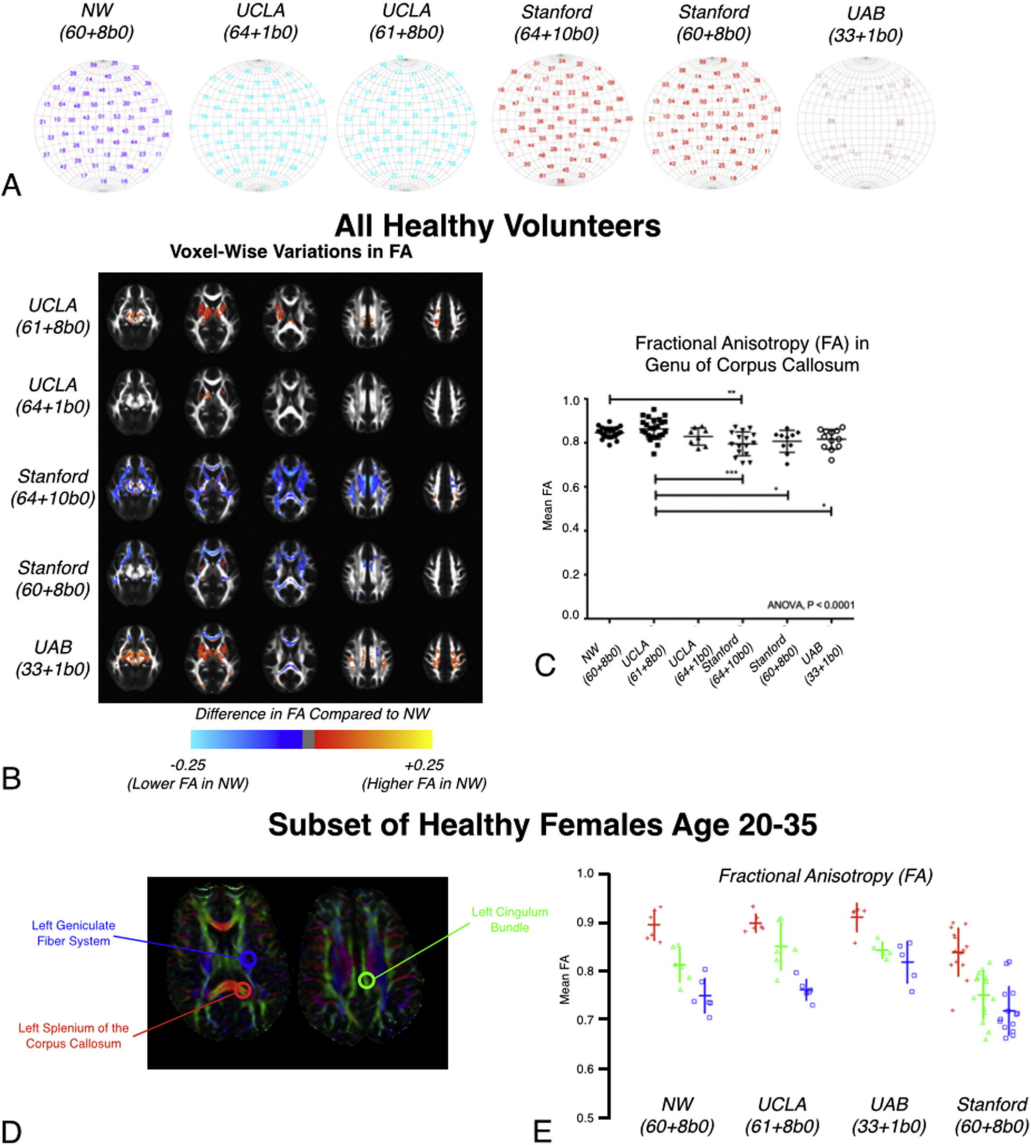
DTI results. A) Visualization of diffusion encoding directions on a sphere for DTI protocols at each site. Note that UCLA and Stanford used two different DTI protocols for MAPP Network neuroimaging. B) Voxel-wise differences in fractional anisotropy (FA) throughout the brain relative to NU (Siemens 3 T Trio, 60 directions + 8 *b* = 0 s/mm^2^) in healthy volunteers. C) Mean FA measurements in the genu of the corpus callosum demonstrating significant differences across sites. D) Regions of interest (ROIs) for major fiber tracts were selected for between-site comparison of FA in the cohort of normal healthy females age 20–35: the left geniculate fiber system (blue) left splenium of the corpus callosum (red) and left cingulum bundle (green). E) Comparison of mean FA measurements from the ROIs illustrated in (D) stratified by site. Results show significant site differences in the corpus callosum (red ROI, *H* = 13.17, *P* = 0.0043), cingulum bundle (green ROI, *H* = 17.30, *P* = 0.0006), and geniculate fibers (blue, ROI) *H* = 13.00, *P* = 0.0046). (For interpretation of the references to color in this figure legend, the reader is referred to the web version of this article.)

Despite efforts to standardize DTI image acquisition across sites, a total of 6 different acquisition protocols were employed during the MAPP Neuroimaging Study (Fig. 4A). Support from the MRI scanner manufacturer (GE, Philips, and Siemens) and experts at each site were required to resolve differences in scanner capabilities and configuration across sites. Many of the sites used 60 diffusion sensitizing directions with *b* = 1000 s/mm^2^ and 8 reference *b* = 0 s/mm^2^ images (60 + 8b0, NU + Stanford), while other sites used a comparable number of directions and reference images including 64 directions and 1 reference image (64 + 1b0, UCLA), 61 directions and 8 reference images (61 + 8b0, UCLA), 64 directions and 10 reference images (64 + 10b0, Stanford). Only UAB was not able to acquire DTI data with more than 32 directions and instead acquired data with 32 directions and a single reference image (32 + 1b0). However, all sites, except one, achieved compatibility during the course of the study. The Neuroimaging Working Group agreed on a standard DTI sequence that could be implemented at every site.

Significant spatial variations in FA and ADC were observed in statistical parameter maps for all healthy volunteers and UCPPS and positive control participants with adequate quality DTI when evaluated between sites. As shown in Fig. 4B, many deep GM and connecting WM structures demonstrated significantly higher or lower FA compared with NU. When examining a 4 mm spherical ROI placed in the genu of the corpus callosum, results demonstrated significant differences in FA (Fig. 4C; ANOVA, *P* < 0.0001). Tukey's test for multiple comparisons showed that NU and UCLA (61 + 8b0 protocol) had comparable FA in this region, while UCLA (61 + 8b0 protocol) had significantly higher FA compared with Stanford (both protocols) and UAB. Importantly, the variability in FA measurements within the corpus callosum was substantially lower in the NU dataset compared with other sites.

Lastly, DTI measurements from a 9 pixel ROI placed in the left splenium of the corpus callosum, left geniculate fiber system, and left cingulum bundle in a subset of healthy female volunteers ages 20–35 were examined (Fig. 4D). Significant differences in FA (Fig. 4E; Kruskal–Wallis, *P* < 0.05), ADC, AD, and RD were observed across sites.

## 4 Discussion

### 4.1 Overview of trans-MAPP neuroimaging

The goal of the trans-MAPP Neuroimaging Study was to obtain multimodal MRI neuroimaging data by combining 3D T1-weighted structural, rs-fMRI and DTI data from a large cohort of participants with UCPPS and healthy and “positive” control study participants, and integrate those data with clinical and epidemiological information, including biomarker assays of bio-specimens. The primary objective of the MAPP Network neuroimaging effort was to identify the structural/functional brain features implicated in UCPPS that can inform integrated clinical phenotyping efforts in the MAPP Network and ultimately inform on future clinical studies (e.g., trial design) and clinical management (e.g., direct individualized treatments toward phenotypic sub-groups). Even though it may not be practical to use brain-based biomarkers in the clinic or in clinical trials, they are important in validating symptom based patient subtypes that respond to different types of therapies. In this regard, the MAPP Network attempted to emulate the examples established by other pioneering neuroimaging research networks, including the International Consortium for Brain Mapping (ICBM) (Mazziotta et al., 2001), ADNI (Jack et al., 2004), fBIRN (Glover et al., 2012), NIH Pediatric MRI Database (Evans, 2006), the Autism Brain Imaging Data Exchange (ABIDE) (Di Martino et al., 2014), PharmaCog (Jovicich et al., 2013), and HIV Neuroimaging Consortium (HIVNC) (Chang et al., 2004, Dewey et al., 2010). The MAPP Network faces the additional challenge of identifying subjects with distinct brain signatures in a highly heterogeneous clinical population that likely encompasses multiple underlying disease states. As one of the core symptoms of UCPPS, chronic pain is notorious for its subtle and variable anatomical brain changes that impact local and global brain activity (Farmer et al., 2011, May, 2011, Schmidt-Wilcke, 2008).

Pooling of UCPPS imaging data to facilitate detection of clinical subgroups is a central network goal. To support this objective, the present study examined the presence and extent of between-center differences for each neuroimaging modality. This investigation focused on the MAPP Network UCPPS population as a whole, as well as a more homogenous subgroup of female healthy control participants. The analysis strategies included several of the quality control methods described by Huang (Huang et al., 2012) that were paired with additional novel analytic approaches. The primary finding of the present study is the identification of significant between-center differences in all three types of neuroimaging data and these differences persisted even when age and gender were accounted for. Below we consider the potential causes and consequences of these center differences have for neuroimaging-based clinical phenotyping in UCPPS.

### 4.2 Possible explanations for observed site differences

In multi-site studies a prominent potential source of between-site bias can proceed from an imbalance in the distribution of subjects, such as differing numbers of men and women, age groups or participant cohorts. However, one set of analyses performed in a highly homogeneous group (healthy control females aged 20–35 years) also showed statistically significant site differences, suggesting that distribution of subjects at sites may not be a likely source for the inter-site differences we observed. Furthermore, another set of analyses specifically controlled for age and gender. Other possible causes include differences between scanner hardware/software platforms and the use of different image acquisition parameters and/or procedures. There is a growing body of literature suggesting that MRI scanners produced by different manufacturers, as well as different scanner models built by a single manufacturer, can produce significantly different measurements (Abdulkadir et al., 2011, Bendfeldt et al., 2012, Clarkson et al., 2009, Friedman and Glover, 2006a, Friedman and Glover, 2006b, Friedman et al., 2006, Kruggel et al., 2010, Reig et al., 2009, Saotome et al., 2012, Stonnington et al., 2008, Takao et al., 2012, Yendiki et al., 2010). Authors of several of these studies have stated that the between-scanner differences were small compared to differences produced by disease (Abdulkadir et al., 2011, Stonnington et al., 2008) or normal aging (Bendfeldt et al., 2012, Evans, 2006, Kruggel et al., 2010). However, given that chronic pain populations are often distinguished by subtle brain changes (May, 2011, Schmidt-Wilcke, 2008) such small differences may inhibit detection of disease-specific biomarkers for transition to pain chronification, pain maintenance, and predisposition to develop chronic pain. Conversely, if significant differences in imaging changes are observed, they should be considered robust enough to survive the across-site differences and associated noise.

### 4.3 Site differences in structural MRI measurements

Across site evaluations as part of the MAPP Neuroimaging Study suggest Philips MRI systems tended to produce lower GM volume estimates when compared with Siemens or GEMS systems. This may be attributable to the reduced digital resolution that Philips systems use to represent MR signal intensity. Low digital resolution reduces the accuracy of distinguishing small signals of background, CSF and GM and this may have resulted in the failure of intensity normalization and brain extraction and therefore to low measures of GM volume. This hypothetical picture is supported by evidence of reduced low signal contrast in ADNI and fBIRN phantom images (Fig. 1). Moreover fuzzy c-means tissue segmentation produced ‘false’ CSF assignments in cortical gray matter more frequently for PHC 3D-T1-MRI images compared with those collected by Siemens and GEMS scanners. The Philips scanner at Michigan produced the lowest GM volumes. It is hypothesized that this bias resulted from a combination of reduced Philips digital resolution combined with the fact that the University of Michigan consistently used a voxel size that was approximately 35% smaller than used at the other centers. These observations underscore the importance of not only acquiring high quality images, but also considering how post processing algorithms interact with acquired data.

In addition to the between-manufacturer differences, within manufacturer differences were also observed. The Siemens scanners at NW and UCLA were nearly identical, yet phantom and human studies suggested these two MRI units have intrinsically different SNR performance, which may reflect the subtle differences observed between NW and UCLA GM volumes.

### 4.4 Site differences in rs-fMRI measurements

rs-fMRI protocols were well harmonized across MAPP Network scanning sites and were modeled after previous fBIRN work (Friedman and Glover, 2006a, Friedman and Glover, 2006b, Friedman et al., 2006, Glover et al., 2012, Magnotta and Friedman, 2006). Nevertheless, site differences in fBIRN QC parameters (SNR and SFNR) were still observed. These site differences may be due to intrinsic SNR and digital resolution issues that have been described above. SFNR may be sensitive to the basic performance of several major MRI subsystems including the radiofrequency transmit and the gradient systems. The fact that two nearly identical Siemens systems (NW and UCLA) produced consistently different SFNR and SNR reinforces that rs-fMRI performance may be dependent on technological factors other than manufacturer.

### 4.5 Site differences in DTI measurements

Performance differences in FA within the MAPP Network data were observed, in agreement with other multicenter studies (Fox et al., 2012, Zhu et al., 2011), despite FA being the most reproducible DTI parameter across sites (Fox et al., 2012, Jovicich et al., 2014, Vollmar et al., 2010). The precise mechanisms that underlie the differences are currently under investigation; however, it is conceivable that the combination of different diffusion sensitizing gradient schemes, SNR performance, image filtering, and number of *b* = 0 s/mm^2^ images may be responsible for in site-dependent differences in DTI metrics.

### 4.6 Impact of site differences in pooled analyses

Pooling multicenter neuroimaging data has clear advantages over single center study data, including increased sample sizes needed to identify clinical subgroups, greater statistical power, and the potential to rapidly advance clinical phenotyping and treatment. The presence of scanner-related differences does not imply the data collected for the MAPP Network cannot be pooled. The statistical impact of pooling depends not only on the type and magnitude of technological differences but also on effect size and the number of subjects studied. For example, if biological effect sizes are similar to or greater than between-center or scanner-related differences, pooling may still have value but may require careful statistical corrections. In contrast, the present study demonstrates that pooling of multimodal imaging data will likely require appropriate statistical corrections to account for site and/or scanner effects that may otherwise obscure clinically meaningful features of brain function and structure. For example, differences in FA measurements were significant, but small (< 0.1) relative to expected biologic changes (0.1–0.2) when comparing healthy control data between NW and UCLA, suggesting site differences may be largely ignored when combining the data (Fig. 4A). On the other hand, the Stanford 64 + 10b0 DTI protocol, which was markedly different, showed site differences on the order of those expected biologically (~ 0.2), indicating that site corrections has to be considered when using these data. Similar arguments can be made regarding fMRI and structural data, where differences in image quality between sites may provide less sensitivity to patient/control differences. Thus, there is a need to balance the increased statistical power obtained by increasing patient numbers while also being mindful of and correcting for site differences. It is notable that several authors have concluded that scanner-related bias is small compared to age, gender and disease changes (see above), yet most disease-related neuroimaging work has focused on populations with overt neurologic and/or psychiatric disease characterized by gross brain abnormalities. In contrast, the brain changes associated with disease processes that underlie UCPPS or other chronic pain conditions may be subtle such that effects could be overwhelmed by scanner or site-related biases.

### 4.7 Between-site harmonization

Clinical and research MRI centers use a wide variety of unique MRI hardware/software system configurations. Accordingly the MAPP Network sought to develop a practical cross-platform multimodal image acquisition protocol. Practical strategies were used to harmonize image acquisition parameters and procedures across five academic biomedical research centers using eight different MRI hardware/software systems produced by the three major MRI systems manufacturers. The primary approach used to achieve harmonization was to encourage centers to use a consistent set of forward-prescribed image acquisition parameters, while respecting each center's capabilities, limitations, and preferred protocols. The initial image acquisition parameters and procedures were agreed upon in a series of preliminary teleconferences in which technical experts from each center participated. Prior to study initiation, phantom imaging was used to assess the quality of each center's data and procedures. Lastly, image quality control was assured through both qualitative and quantitative assessments of images acquired and subsequently uploaded into the MAPP Network neuroimaging database. This process ensured all investigators using MAPP Network neuroimaging data were using the highest quality data available.

The MAPP Network encountered several problems with achieving optimal protocol harmonization. These problems arose from the following factors, given in order of significance: 1) Scanners made by different MRI manufacturers are intrinsically different. For example, the network investigators did not anticipate that digital resolution in 3D T1-weighted structural MR images produced by Philips MR systems would be further compromised by the use of small voxel sizes at one center. Similarly, the investigators were unable to address manufacturer differences in DTI acquisition software and procedures. Furthermore, there are manufacturer-specific differences in fMRI image reconstruction as has been described by Friedman and Glover (Friedman and Glover, 2006a, Friedman and Glover, 2006b, Friedman et al., 2006). 2) As is the case with most large-scale studies, human error contributed to acquisition and procedural deviations that had to be identified through quality control assessment. 3) The expected and inevitable upgrades occurred and these may have contributed to some of the between-center variability.

Despite subtle differences in neuroimaging measurements between sites, the MAPP Network has successfully used various statistical techniques (see references listed in this paragraph) to correct for these site differences and demonstrate both functional and structural changes within the brain due to chronic pelvic pain using the combined, multi-site database. The MAPP Network has published 7 original manuscripts using multisite neuroimaging data demonstrating these successes (Bagarinao et al., 2014, Farmer et al., 2015, Kairys et al., 2015, Kilpatrick et al., 2014, Kutch et al., 2015, Martucci et al., 2015, Woodworth et al., 2015). For example, a recently published MAPP Network study by Bagarinao et al. (2014) used a multivariate pattern classification approach to detect structural changes in brain morphology associated with chronic pelvic pain, noting significant cortical gray matter changes in the primary somatosensory cortex, pre-supplementary motor area, hippocampus, and amygdala consistent with previous studies in visceral pain syndromes. This study used 33 female UCPPS participants and 33 age- and gender-matched healthy control subjects selected from all 5 sites. Similarly, a published MAPP Network study by Kairys et al. (2014) examined UCPPS females and noted significantly increased gray matter volume in the primary somatosensory cortex, superior parietal lobe, and supplemental motor area, also noting correlation of gray matter volume with pain, mood, and urological symptoms. This study used the same dataset as Bagarinao et al. A recent MAPP Network functional MRI study by Kilpatrick et al. (2014) demonstrated alterations in the frequency distribution in viscerosensory, or posterior insula, somatosensory, and supplementary motor regions in female UCPPS participants compared with healthy controls, implying that women with these conditions have alterations in connectivity within cortico-cerebellar networks previously shown to be associated with bladder function This study utilized all female UCPPS participants (82) and healthy control participants (85) from all 5 sites that underwent neuroimaging as part of MAPP Network protocols. In addition, a MAPP Network functional MRI study by Kutch et al. (Kutch et al., 2015) illustrated altered motor control activity and connectivity specific to the pelvic floor in male UCPPS participants. So far two studies have been published that utilized the DTI data to evaluate differences in UCPPS subjects. Farmer et al. looked at skeletonized FA values in female subjects and found distributed regions of decreased FA as well as increased in FA in superior brain regions and inferior regions associated with intra-hemispheric white matter fibers; these some of these regions of different FA correlated with symptom scores (Farmer et al., 2015). Woodworth et al. looked at voxel-wise changes in traditional DTI values, such as FA and mean diffusivity (MD), as well as advanced and higher-order diffusion MRI metrics, such as track density imaging (TDI) and generalized anisotropy (GA), and found global decreases in FA, GA and TDI, as well as global increases in MD, and select regions presented with increases in FA and GA; the mean values of these metrics showed trends of correlations with symptom scores and disease duration (Woodworth et al., 2015). Together, these studies clearly demonstrate how increased statistical power resulting from large multicenter populations within the MAPP Network, combined with appropriate statistical corrections, can be used to overcome significant site differences likely arising from slight differences in acquisition parameters and hardware specifications. Numerous analyses of the rich, multi-site neuroimaging data, including stratifications using urologic and non-urologic measures and incorporation of results from other, integrated MAPP Network studies (e.g., biomarker efforts, quantitative sensory testing, etc.) are ongoing and are expected to allow for an unprecedented insight into the pathophysiology of UCPPS.

### 4.8 Correction methods implemented in MAPP manuscripts and theoretical framework for multi-site correction

In order to account for site differences in the analysis of multi-site data the main technique available to researchers involves measuring the mean across sites and normalizing. This can either be done either with resultant values derived from ROIs or with voxel-wise approaches, or can be taken into account in a general linear model (GLM) or similar statistical models. Many of the current neuroimaging MAPP manuscripts have taken this approach. Bagarinao et al. used data from all five sites, and implemented an additional layer of visual quality assessment; for regression of site effects of GM intensity, they applied a voxel-wise correction factor determined by the mean intensity of the voxel, divided by the mean GM intensity of voxels for that site, minus the mean intensity of GM voxels for all sites (Bagarinao et al., 2014). Farmer et al. used a similar correction method for their data, using FA values derived from DTI as the measure of interest, but employing a normalization with respect to the site mean and the global mean of the study by subtracting the patients mean from the mean of the site, and added this to the global mean to obtain a normalized FA value (Farmer et al., 2015). For the frequency analysis performed in their study, Kilpatrick et al. implemented a flexible factorial analysis in SPM that accounted for site, and in the subsequent functional connectivity analysis performed from the significant clusters in the frequency analysis they used site as a covariate (Kilpatrick et al., 2014).

Site differences are heavily dependent on the analysis being performed: for some analyses that use larger ROIs or robust post-processing tools, correction for site may not be strictly necessary. For example, Kairys et al. analyzed a particular gray matter region (primary sensory cortex) and found no significant differences across sites for volume or TIV (which is a sum of the value of GM, WM, and CSF over the whole brain), in either the ANOVA analysis or GLM analyses they performed (Kairys et al., 2015). Martucci et al. performed an ICA analysis and a seed-based connectivity analysis, and to verify that site differences did not cause sufficient effects to affect the analysis, they performed supplemental tests with regression of site and found virtually the same results as the analysis performed without site regression (Martucci et al., 2015). For some analyses selective use of particular imaging data based on homogeneity of scanner and sites can be useful. For example, Kutch et al. selected male patients from NW and UCLA for their rs-fMRI analysis, and when evaluating differences across sites found no difference across sites in both the ANOVA analysis and in a post-hoc multiple comparisons test (Kutch et al., 2015). Similarly, Woodworth et al. opted to use subject scans from NW and UCLA data for their DTI analysis, given that they used advanced fiber-tracking techniques and computed higher order diffusion metrics such as GA, and they included site as a covariate in their model and found only minor differences between sites in a supplemental analysis (Woodworth et al., 2015). As can be seen by the range of approaches to site correction outlined in these manuscripts, they are dependent on the level and type of analysis performed, and thus the best directions for correcting for sites will be highly dependent on the type of analysis performed.

### 4.9 Recommendations for the future

Several recent studies (published after the MAPP Network was initiated) focused on calibration methods to make data generated by different MRI scanners more homogeneous (Clarkson et al., 2009, Maikusa et al., 2013, Marchewka et al., 2014, Ribbens et al., 2014), and these methods provide valuable guidance to achieve better harmonization. Furthermore, once the technical cause(s) of scanner/center-related bias are understood (e.g., SNR differences, digital resolution differences, gradient table differences, or subtle differences in acquisition parameters), it is possible to develop pre-specified (or post hoc) approaches that address the contributing factors. Ongoing evaluations of technical confounds and biases continue to clarify such factors in the MAPP Network data.

Longitudinal collection of a complex multiparametric image acquisition procedure necessitates frequent and detailed quality control pipelines. Given the inevitable biases in multicenter data, investigators who plan to embark on multicenter neuroimaging projects should consider that core imaging laboratory personnel, equipped with software systems designed to perform rapid turnaround QC, are critical assets for establishing initial harmonization and sustaining it longitudinally (Seibyl et al., 2010) Equally important is the identification of a technical expert at each center, such as a senior technologist or MR physicist, who is knowledgeable about their center's MRI equipment and can make changes to acquisition parameters when needed.

Moving toward the future, these changes have been implemented for the phase II of the MAPP study. Improvements in scanner technology, further development of standardized multisite protocols, and customized collaboration methods were developed as part of the MAPP I Network Study. The imaging protocols for each modality have been standardized to provide the highest common denominator scans for this longitudinal multi-site study. An automated QC pipeline to check DICOM parameters has been implemented and semi-automated quality control of incoming data will be performed (Labus et al., 2015). Coordination between the data collection, neuroimaging, and governance committees has led to a cohesive approach for further imaging studies. It is expected that this unprecedented collaborative neuroimaging effort implemented in MAPP I will lead to a better understanding of UCPPS pathophysiology, the identification of neurobiological subgroups, and the identification of risk factors. In addition, the findings from these studies are likely to inform future clinical trials in UCPPS.

## Figures and Tables

**Table 1:** Summary of patient cohorts.

Research group	NW	UCLA	Michigan	Stanford	UAB	Total
Healthy control	25	35	37	19	12	128
Positive control	10	32	17	10	0	69
Fibromyalgia	x	x	x	x		
IBS	x	x	x	x		
CFS	x		x			
UCPPS	24	33	34	24	17	132
Total	59	100	88	53	29	329

**Table 2:** 3D structural MRI acquisition parameters. (Note: There are slight differences between sequence parameters defined for Siemens and GEMS or Philips, including definition of TR and the required flip angle for similar CNR).

Institution name	NW	UCLA	Michigan	Stanford	UAB
Scanner manufacturer	Siemens	Siemens	Philips	GEMS	Philips
Scanner model	Trio Tim	Trio Tim	Ingenia	Discovery MR750	Achieva
Software version	B17	B15	4.1.1–4.1.2	DV22.0	2.6.3
Field strength (T)	3	3	3	3	3
Acquisition type	3D	3D	3D	3D	3D
Image orientation	Axial obl	Axial obl	Axial obl	Axial obl	Axial obl
Flip angle [degrees]	9	9	8	11	9
Repetition time (TR) [ms]	2200	2200	6.6–7.1	6.8–7.4	7.1–7.2
Echo time (TE) [ms]	3.3	3.3	4.7	2.8	3.2–4.7
Inversion time (TI) [ms]	900	900	790–850	450	835–844
Number of averages (NEX)	2	2	1	2	1
Pixel bandwidth [Hz]	241	200	246–247	391	241
Field of view (FOV) [mm]	256	256	256	220	256
Acquisition matrix	256 × 256	256 × 256	288 × 288	256 × 256	288 × 288
Slice thickness	1 (0)	1 (0)	0.9 (0)	1 (0)	1 (0)
Voxel resolution [mm]	1 × 1 × 1	1 × 1 × 1	0.9 × 0.9 × 0.9	0.86 × 0.86 × 1	1 × 1 × 1

**Table 3:** Resting-state fMRI acquisition parameters.

Institution name	NW	UCLA	Michigan	Stanford	UAB
Scanner manufacturer	Siemens	Siemens	Philips	GEMS	Philips
Scanner model	Trio Tim	Trio Tim	Ingenia	Discovery MR750	Achieva
Software version	B17	B15	4.1.1–4.1.2	DV22.0	2.6.3
Field strength (T)	3	3	3	3	3
Acquisition type	2D EPI	2D EPI	2D EPI	2D EPI	2D EPI
Image orientation	Axial obl	Axial obl	Axial obl	Axial obl	Axial obl
Flip angle [degrees]	77	77	77	77	77
Repetition time (TR) [ms]	2000	2000	2000	2000	2000
Echo time (TE) [ms]	29	28	30	30	30
Number of repetitions [frames]	10,800	12,000	9000–14,000	9600	9600
Pixel bandwidth [Hz]	2003	3005	2000–2200	7813	3050
Field of view (FOV) [mm]	220	220	220	220	220
Acquisition matrix	64 × 64	64 × 64	64 × 64	64 × 64	64 × 64
Slice thickness (gap) [mm]	4 (0.5)	4 (0.5)	4 (0.5)	4 (0.5)	4 (0.5)
Voxel resolution [mm]	3.44 × 3.44 × 4	3.44 × 3.44 × 4	3.44 × 3.44 × 4	3.44 × 3.44 × 4	3.44 × 3.44 × 4

**Table 4:** Diffusion tensor imaging acquisition protocol.

Institution name	NW	UCLA 1	UCLA 2	Michigan	Stanford 1	Stanford 2	UAB
Scanner manufacturer	Siemens	Siemens	Siemens	–	GEMS	GEMS	Philips
Scanner model	Trio Tim	Trio Tim	Trio Tim	–	Discovery MR750	Discovery MR750	Achieva
Software version	B17	B15	B15	–	DV22.0	DV22.0	2.6.3
Field strength (T)	3	3	3	–	3	3	3
Acquisition type	2D EPI	2D EPI	2D EPI	–	2D EPI	2D EPI	2D EPI
Image orientation	Axial obl	Axial obl	Axial obl	–	Axial obl	Axial obl	Axial obl
Flip angle [degrees]	90	90	90	–	90		90
Repetition time (TR) [ms]	9500	9400	9500	–	9600	9000	14,527–14,686
Echo time (TE) [ms]	88	87	88	–	93–94	76–90	75–76
*b*-Values [# acq] [s/mm^2^]	0/1000 (8/60)	0/1000 (1/64)	0/1000 (8/61)		0/1000 (10/64)	0/1000 (8/60)	0/1000 (1/33)
Number of directions (*b* > 0)	60	64	61	–	64	60	33
Pixel bandwidth [Hz]	1347	1630–1700	1630–1700	–	1953	1953–3906	1784–1786
Field of view (FOV) [mm]	256	256	256	–	220	256	220
Acquisition matrix	128 × 128	128 × 128	128 × 128	–	128 × 128	128 × 128	128 × 128
Slice thickness (gap) [mm]	2 (0)	2 (0)	2 (0)	–	4 (0.5)	2 (0)	2 (0)
Voxel resolution [mm]	2 × 2 × 2	2 × 2 × 2	2 × 2 × 2	–	1.6 × 1.6 × 4	2 × 2 × 2	1.6 × 1.6 × 2
